# Prevalence and Assessment of Malnutrition Using Nutritional Indices and Body Mass Index in Relation to Coronary Artery Disease in Japanese Patients: A Retrospective Real-World Study

**DOI:** 10.7759/cureus.90738

**Published:** 2025-08-22

**Authors:** Jennifer Saulam, Fumiaki Mikami, Kazushi Murakami, Jacqueline Saulam, Joseph Kuriakose, Tetsuo Minamino, Hideto Yokoi

**Affiliations:** 1 Department of Medical Informatics, Faculty of Medicine, Kagawa University, Kagawa, JPN; 2 Department of Food Processing and Nutrition, Karnataka State Akkamahadevi Women's University, Karnataka, IND; 3 Department of Cardiorenal and Cerebrovascular Medicine, Faculty of Medicine, Kagawa University, Kagawa, JPN; 4 Department of Radio Diagnosis, Morning Star Medical Centre, Kerala, IND; 5 Department of Respiratory Medicine, Morning Star Medical Centre, Kerala, IND

**Keywords:** body-mass index, cardiovascular risk, malnutrition prevalence, nutritional indices, real-world data

## Abstract

Background

Cardiovascular disease (CVD) remains a leading cause of mortality worldwide, with a disproportionate burden in low- and middle-income countries (LMICs). While malnutrition is a recognized clinical issue, its role as a contributing factor to coronary artery disease (CAD) is often overlooked. This study aimed to assess the prevalence of malnutrition and its association with significant coronary artery stenosis (CAS) using routinely available nutritional indices, in combination with body mass index (BMI), in a real-world Japanese patient cohort.

Methods

This retrospective cross-sectional study included 1,107 patients who underwent coronary angiography (CAG) at Kagawa University Hospital, Kagawa, Japan, between January 2006 and December 2023. Nutritional status was assessed using various indices: controlling nutritional status (CONUT), prognostic nutritional index (PNI), and geriatric nutritional risk index/nutritional risk index (GNRI/NRI). Malnutrition was defined using standard clinical thresholds. CAS was defined as 75% or greater luminal narrowing in at least one coronary artery. Multivariable logistic regression models were developed to assess the association between nutritional indices and CAS, adjusting for age, sex, smoking status, and lipid levels. Interaction terms were included to evaluate the modifying effect of BMI (continuous). Additional risk phenotypes were created by combining nutritional indices and BMI based on the 75th percentile.

Results

Significant CAS was identified in 45.4% of the study population. Malnutrition prevalence using standard clinical cutoffs ranged from 26.8% using CONUT to over 70% with GNRI/NRI and PNI, with poor inter-index agreement (Fleiss’ κ = 0.227). Interaction analysis showed that severe-risk GNRI/NRI scores were positively associated with CAS at higher BMI levels, while mild-risk scores showed inverse associations. Several phenotype-based combinations were also significantly associated with increased CAS risk, including high CONUT + normal BMI (Odds ratio, OR = 1.88), low CONUT + high BMI (OR = 1.75), and high PNI + high BMI (OR = 1.92). Among predictive models, the CONUT + BMI combination demonstrated the best overall discriminative performance (AUC = 0.71; sensitivity = 78.6%), while the PNI + BMI model had the highest positive predictive value (66.7%).

Conclusion

Malnutrition is prevalent among patients undergoing CAG and is significantly associated with CAS, particularly when assessed in combination with BMI. Our findings suggest that GNRI/NRI, when interacted with BMI as a continuous variable, provides a sensitive gradient of risk, while percentile-based combinations using CONUT or PNI offer clear categorical phenotypes for risk stratification. These accessible, routinely used biomarkers and EMR-based indices can serve as practical tools for early risk identification, especially in resource-limited LMIC settings. Prospective studies are warranted to determine whether targeted nutritional interventions based on these indices can improve cardiovascular outcomes.

## Introduction

Cardiovascular disease (CVD) remains the leading cause of mortality worldwide, responsible for approximately 17.9 million deaths in 2019, accounting for 32% of all global deaths. Alarmingly, over 75% of these occur in low- and middle-income countries (LMICs), highlighting significant global health disparities [1,2]. In Japan, despite declining age-standardized mortality rates for both stroke and ischemic heart disease over recent decades, CVD continues to impose a substantial public health burden, particularly among the aging population [1]. Early identification of individuals at elevated risk is crucial for prevention, yet healthcare systems in LMICs often lack access to advanced imaging, comprehensive medication records, and specialized laboratory diagnostics. This underscores the urgent need for simple, scalable, and cost-effective screening tools based on readily available clinical and laboratory data [3,4].

Nutritional status has emerged as a critical, yet often overlooked, determinant of cardiovascular health. Malnutrition, including both undernutrition and nutrient deficiencies that may occur even in individuals with normal or elevated body mass index (BMI), is increasingly recognized for its role in promoting systemic inflammation, metabolic dysregulation, and accelerated atherosclerosis. Despite its clinical significance, routine nutritional assessment remains underutilized in cardiovascular care, particularly in settings with constrained resources [5-7].

Several objective indices have been developed to assess nutritional status using standard clinical and biochemical parameters such as serum albumin, total cholesterol, lymphocyte count, body weight, and height. Among these, the controlling nutritional status (CONUT) score [6-8], prognostic nutritional index (PNI) [6,7,9], nutritional risk index (NRI) [7,10], and geriatric nutritional risk index (GNRI) [6,11] have gained growing attention in cardiovascular medicine for their simplicity and prognostic utility. These indices are inexpensive to calculate, require no advanced equipment, and have demonstrated predictive value in various clinical domains, including oncology, surgery, and, increasingly, cardiology [5-7,12,13].

In Japanese contexts, the prevalence and impact of malnutrition are appreciably high. For example, Ishizu et al. found that among older Japanese patients undergoing transcatheter aortic valve implantation, between 14% and 61% had moderate-to-severe malnutrition depending on the index (CONUT, PNI, or GNRI), with nearly 90% classified as malnourished by at least one metric [14]. Community-based data further reveal that a significant proportion of older Japanese adults receiving home care is undernourished or at risk [15], and national surveys consistently register undernutrition in aging cohorts [16]. These findings highlight the importance of integrating BMI with multiple nutritional indices, reflecting muscular, metabolic, and immunonutritional aspects, to enhance cardiovascular risk stratification across diverse healthcare settings, from high-resource countries like Japan to resource-limited regions, using universally accessible, cost-effective, and scalable data [14-16].

However, these tools assess different physiological dimensions, ranging from immune function to protein-energy balance and therefore may not consistently classify individuals into the same nutritional risk categories [5,6]. Serum albumin is the only parameter common across all indices, and its variability can significantly influence outcomes [13,17]. The real-world concordance among these indices in cardiovascular populations remains poorly understood [7]. Additionally, BMI, while widely used, fails to distinguish between lean and fat mass, and may overlook conditions such as sarcopenic obesity, which is especially prevalent in older or metabolically unhealthy individuals [18-20].

Given these limitations, integrating BMI with nutritional indices may yield a more nuanced understanding of cardiovascular risk. This is particularly relevant in LMICs, where the double burden of undernutrition and overnutrition coexists, and where clinical decisions must often be made with limited diagnostic information [21-22].

To address these gaps, we conducted a retrospective study using electronic medical record (EMR) data from a Japanese hospital, purposefully focusing on basic clinical parameters that simulate a resource-constrained environment. Although conducted in a high-income country, the use of Japanese EMR data, limited to universally available variables, offers a pragmatic model to emulate the diagnostic constraints faced in LMICs, enhancing the external validity and applicability of our findings.

This study aimed to estimate the prevalence of malnutrition, as defined by CONUT, PNI, and GNRI/NRI, in patients undergoing coronary angiography (CAG). It also assessed the association of these nutritional indices, individually and in combination with BMI, with significant coronary artery stenosis (CAS).

By leveraging universally accessible data, this study aims to inform practical, low-cost, and scalable cardiovascular risk assessment strategies, applicable across both high-resource and low-resource healthcare systems.

## Materials and methods

Study design and population

This retrospective, cross-sectional study utilized real-world data obtained from coronary angiography (CAG) reports and EMRs at Kagawa University Hospital, Kagawa, Japan, spanning from January 2006 to December 2023.

During the study period, a total of 7,860 individuals were registered with CAG identification numbers. To ensure analytical independence and avoid duplication, only the first CAG per patient was included in the analysis. Eligible patients were adults aged 18 years or older and had complete data available for height, weight, serum albumin, total cholesterol, and lymphocyte count.

**Figure 1:**
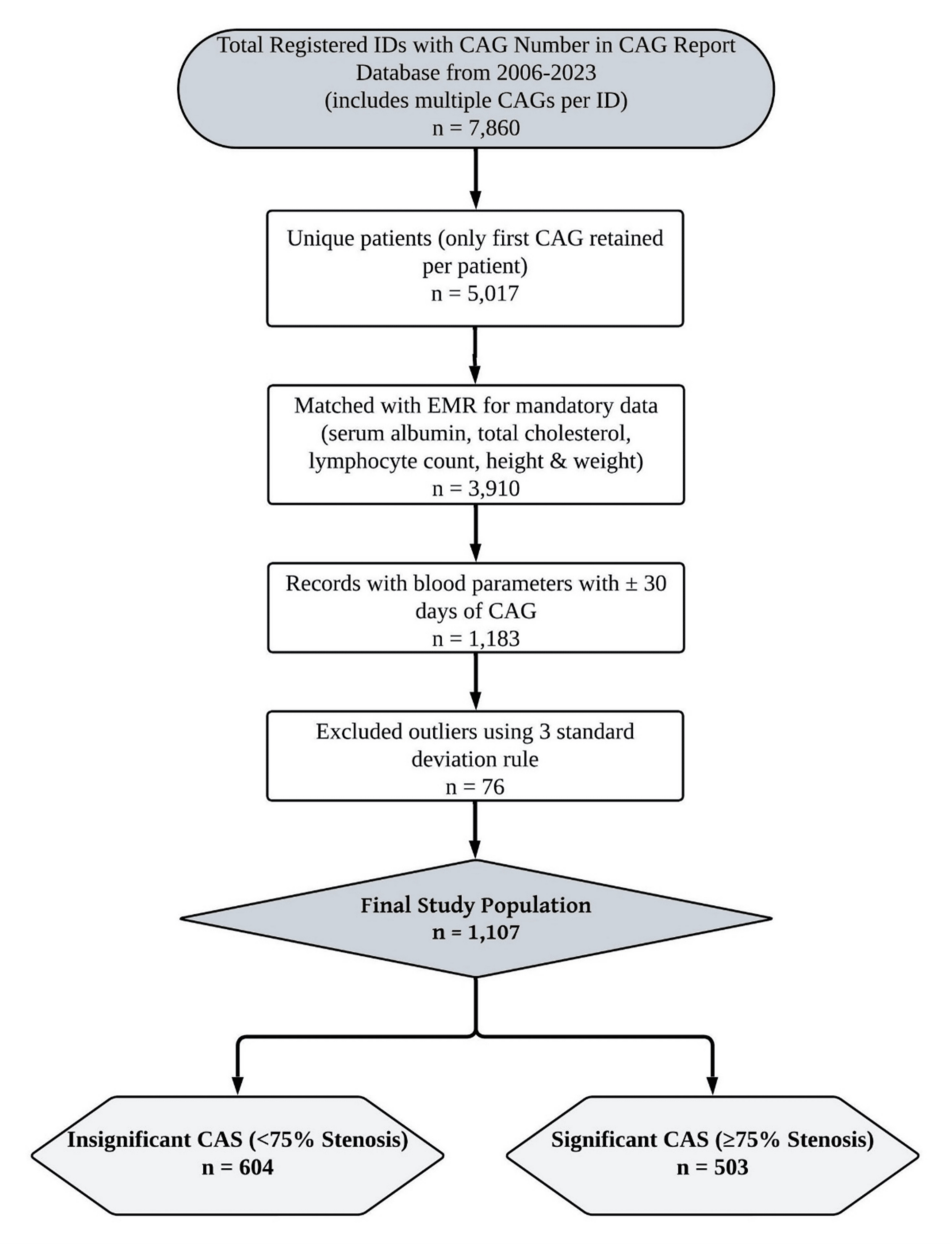
Patient selection flowchart CAG: Coronary angiography; CAS: Coronary artery stenosis; EMR: Electronic medical records.

Patients were excluded if they were pregnant or lactating. While no patients were excluded based on the presence of fluid collections such as ascites or pleural effusion, only serum-based parameters were utilized to minimize bias in weight-dependent calculations. Records with incomplete or unmatched CAG and laboratory data, as well as those containing outlier values beyond three standard deviations, were also excluded. Following the application of inclusion and exclusion criteria, a total of 1,107 unique patients were included in the final analysis. The patient selection process is illustrated in Figure 1.

This study was approved by the Research Ethics Committee of Kagawa University, Faculty of Medicine, Kagawa, Japan (Reference No. 2023-211). In accordance with university guidelines, study details were disclosed on the institutional website, and no participants opted out. All data were anonymized prior to analysis, and the study was conducted in accordance with the principles of the Declaration of Helsinki.

Classification of CAS

Significant CAS was defined as a luminal diameter reduction of ≥75% in at least one major coronary artery, as determined from formal coronary angiography (CAG) reports, in accordance with established clinical definitions and previous literature [23]. Of the 1,107 patients, 503 had significant CAS (45.4%), and 604 had insignificant CAS (54.6%).

Assessment of nutritional status and BMI

Nutritional status was evaluated using four validated indices: the controlling nutritional status (CONUT) score, developed by de Ulibarrí et al. [8]; the NRI, originally described by Buzby et al. [10]; the GNRI, a modified version of the NRI developed by Bouillanne et al. [11]; and the PNI, simplified by Onodera et al. [9]. Each of these indices was calculated using routine laboratory or anthropometric data, with the respective formulas and categorization strategies summarized in Table 1. Where specific categorization thresholds were not explicitly provided in the original publications, particularly for NRI and PNI, cutoffs and classification schemes were adopted from a previous study by Roubín et al. [7], who presented a structured overview of formulas, scoring, and cutoffs for CONUT, NRI, and PNI (see their image 1A). Categorization for GNRI was derived directly from the original paper by Bouillanne et al. [11], which clearly defined four nutritional risk categories.

**Table 1:** Formula and categorization strategy for the evaluation of each nutritional index

Based on the standard cutoff points						Based on the 75^th^ percentile		
						Score	BMI classification	Combined categories
Controlling nutritional status (CONUT)								
Categorization	Components	No risk (0-1)	Mild risk (2-4)	Moderate risk (5-8)	Severe risk (9-12)	Low score: <75th percentile. High score: ≥75th percentile	Underweight: <18.5. Normal: 18.5-24.9. High: ≥25	(1) Low + Underweight BMI, (2) Low + Normal BMI, (3) Low + High BMI, (4) High + Underweight BMI, (5) High + Normal BMI, and (6) High + High BMI
Score	Albumin, g/dL	≥3.5 (0)	3.0-3.4 (2)	2.5-2.9 (4)	<2.5 (6)			
	Total cholesterol, mg/dL	≥180 (0)	140-179 (1)	100-139 (2)	<100 (3)			
	Lymphocyte count, × 10^9^/L	≥1.60 (0)	1.20-1.59 (1)	0.80-1.19 (2)	<0.80 (3)			
Formula	Albumin score + Total cholesterol score + Lymphocyte score							
Nutritional risk index (NRI) [For individuals aged below 65 years]								
Categorization		No risk (≥100)	Mild risk (97.50-99.99)	Moderate risk (83.50-97.49)	Severe risk (<83.50)	Same as above	Same as above	Same as above
Formula	(1.519 × Serum albumin (g/L)) + (41.7 × Present weight/Usual body weight)							
Geriatric nutritional risk index (GNRI) [For individuals aged 65 years and above]								
Categorization		No risk (>98)	Mild risk (92-98)	Moderate risk (82-91)	Severe risk (<82)	Same as above	Same as above	Same as above
Formula	(1.489 × Serum albumin (g/L)) + (41.7 × Present weight/Ideal body weight)							
Prognostic nutritional index (PNI)								
Categorization		No risk (>38)	-	Moderate risk (35-38)	Severe risk (<35)	Same as above	Same as above	Same as above
Formula	10 × Serum albumin (g/dL) + 0.005 × Lymphocyte count (mm^3^)							

The application of these indices was age-specific: NRI was used for patients aged below 65 years, while GNRI, designed for older populations, was applied to those aged 65 years and older. Ideal body weight (IBW) was estimated using the Lorentz formula, a historical anthropometric method commonly used in European clinical practice to account for sex-specific physiological differences. According to this formula, IBW is calculated as:

For men: $\text{Height (cm)} - 100 - \left( \frac{\text{Height (cm)} - 150}{4} \right)$

For women: $\text{Height (cm)} - 100 - \left( \frac{\text{Height (cm)} - 150}{2.5} \right)$

This method was originally employed by Bouillanne et al. [11] in the development of the GNRI and later adopted by Raposeiras-Roubín et al. [7] in their application of the NRI. BMI was computed as weight (kg) divided by height squared (m²).

Each nutritional index was analyzed using three distinct methods: (i) standard clinical categorization into low, moderate, or high nutritional risk; (ii) as interaction terms with BMI treated as a continuous variable; and (iii) dichotomized values based on whether the score was above or below the 75th percentile. The dichotomization strategy was adapted from the approach proposed by Kunimura et al. [12] for CONUT and extended in this study to include NRI, GNRI, and PNI. The use of the 75th percentile as a cutoff for dichotomizing nutritional indices was selected to provide a data-driven threshold that balances sensitivity and specificity in identifying patients at higher nutritional risk within our cohort. This percentile-based cutoff allows stratification tailored to the distribution in our study population, improving the clinical relevance of risk phenotyping compared to arbitrary or solely clinical thresholds. These dichotomized indices were further combined with BMI strata, categorized as underweight, normal weight, and high BMI, to create composite nutritional-metabolic phenotypes, which are detailed in Table 1. Notably, a higher CONUT score or lower GNRI, NRI, or PNI score indicated poorer nutritional status.

This multidimensional approach allowed for a comprehensive assessment of both the independent and combined effects of malnutrition and body composition on the severity of CAS, offering a nuanced understanding of the nutritional and metabolic profiles associated with cardiovascular risk.

Statistical analysis

Baseline characteristics were summarized using descriptive statistics. Continuous variables were reported as medians with interquartile ranges (IQRs), and categorical variables as frequencies and percentages. The normality of continuous variables was assessed using the Shapiro-Wilk test and visual inspection of histograms and Q-Q plots. Since all continuous variables were non-normally distributed, non-parametric tests were employed. The Mann-Whitney U test (Wilcoxon rank-sum test) was used to compare continuous variables between groups, and two-sided p-values were reported. Categorical variables were compared using the chi-square test.

To explore the diagnostic consistency across malnutrition classification tools, Cohen’s kappa statistics were computed for pairwise agreement between CONUT, GNRI/NRI, and PNI scores. Fleiss’ kappa was used to evaluate overall agreement among all three tools. A Venn diagram was used to visualize the overlap in malnutrition classification across the indices, and proportions of shared and unique classifications were calculated. These analyses aimed to assess the level of concordance between nutritional tools and the implications of tool selection in clinical settings.

Univariate logistic regression was performed to explore associations between individual clinical, biochemical, and nutritional variables and the presence of significant CAS. Multivariate logistic regression models were constructed to evaluate the independent effects of nutritional indices on CAS, adjusting for relevant confounding variables, including age, sex, smoking status, and lipid levels. Interaction terms with BMI as a continuous variable were also incorporated into the logistic regression models to assess whether the association between nutritional indices and CAS varied by BMI. Odds ratios (ORs) and corresponding 95% confidence intervals (CIs) were calculated.

Model performance was evaluated using receiver operating characteristic (ROC) curve analysis. Discriminatory power was assessed using the C-statistic (area under the curve, AUC). Sensitivity and positive predictive value (PPV) were also calculated to assess diagnostic performance.

All analyses were performed using JMP Pro 17 (SAS Institute Inc., Cary, NC) and R version 4.4.1 software (R Foundation for Statistical Computing, Vienna, Austria). All p-values were two-sided, and p-values < 0.05 were considered statistically significant.

## Results

Patient characteristics

A total of 1,107 patients were included in the study, of whom 503 (45.4%) had significant CAS, and 604 (54.6%) had insignificant CAS. The cohort comprised 771 males (69.7%) and 336 females (30.3%). Patients with significant CAS were slightly older (median age: 73 vs. 72 years; p = 0.006), predominantly male (p < 0.0001), and had a higher prevalence of smoking (52.4% vs. 47.6%; p = 0.03) compared to those with insignificant CAS. Additionally, patients with significant CAS had higher median height (1.62 vs. 1.60 m; p = 0.03) and weight (60.4 vs. 59.5 kg; p = 0.004) (Table 2).

**Table 2:** Baseline characteristics of patients (n = 1107) Data are presented as n (%) for categorical variables and median (IQR) for continuous variables. Chi-square tests (degrees of freedom = 1 for all categorical variables) were used for categorical comparisons. Mann-Whitney/Wilcoxon two-sided tests were used for continuous variables. Test statistics (Z values) for Mann-Whitney tests are reported with a sign (positive or negative) to indicate the direction of difference. P-values < 0.05 were considered statistically significant. BMI: Body mass index; CAS: Coronary artery stenosis; CONUT: Controlling nutritional status; CRP: C-reactive protein; GNRI: Geriatric nutritional risk index; HDLC: High-density lipoprotein cholesterol; LDLC: Low-density lipoprotein cholesterol; NRI: Nutritional risk index; PNI: Prognostic nutritional index; RBC: Red blood cells; WBC: White blood cells.

	Significant CAS (n = 503)	Insignificant CAS (n = 604)	Missing values	Test statistic	p-value
Age, years	73 (66-80)	72 (63-79)	0	2.52	0.006
Sex, male, n (%)	390 (50.6)	381 (49.4)	0	27.13	<0.0001
Smokers, n (%)	99 (52.4)	90 (47.6)	11	4.56	0.03
Alcohol drinkers, n (%)	140 (45.6)	167 (54.4)	20	0.004	0.9
Weight, kg	60.4 (52.5-69.8)	59.5 (50.3-67.4)	0	2.70	0.004
Height, m	1.62 (1.54-1.67)	1.60 (1.52-1.67)	0	1.92	0.03
Lymphocyte count, /μL	1471 (1111-1841)	1478(1086.5-1876.7)	0	-0.18	0.4
Albumin, g/dL	3.9 (3.4-4.2)	3.9 (3.5-4.3)	0	-0.91	0.2
Total cholesterol, mg/dL	165 (143-191)	173.5 (146-199)	0	-3.07	0.001
CRP, mg/dL	0.15 (0.06-0.6)	0.13 (0.05-0.4)	30	2.30	0.01
HDLC, mmol/L	1.09 (0.88-1.32)	1.19 (0.96-1.45)	127	-4.85	<0.0001
Hemoglobin, g/dL	12.6 (11.1-14.2)	12.8 (11.2-14.1)	1	-0.12	0.5
LDLC, mg/dL	95 (75-121)	99 (79-122)	189	-1.43	0.07
Triglycerides, mg/dL	105 (77-150)	96 (70-136)	21	3.12	0.001
Neutrophil count, /μL	4159(3184.5-5329.5)	3713.5(2919-4765.5)	193	4.05	<0.0001
Platelet count, ×10^4^/μL	20.4 (16.7-25.2)	19.3 (15.2-23.6)	26	3.29	0.0005
RBC count, ×10^4^/μL	416 (364-463)	415 (370.3-462)	0	0.23	0.4
WBC count, ×10^2^/μL	63.4 (51.9-77.8)	59 (48.5-73.3)	11	3.99	<0.0001
Blood glucose, mg/dL	118 (102-151)	108 (99-131.3)	378	4.75	<0.0001
BMI, kg/m^2^	23.5 (21.2-25.9)	23.1 (20.7-25.6)	0	2.18	0.014
CONUT score	2 (1-4)	2 (1-4)	0	1.57	0.058
NRI/GNRI score	94.4(80.4-102.6)	92.3 (48.7-101.3)	0	2.52	0.0006
PNI score	46.5 (40.7-51.3)	46.6 (41.4-51.4)	0	-0.86	0.2

Inflammatory and metabolic profiles in CAS

Patients with significant CAS had elevated inflammatory and metabolic markers, including C-reactive protein (CRP), triglycerides, neutrophil count, white blood cell (WBC) count, and platelet count (all P < 0.05). Conversely, they had significantly lower high-density lipoprotein cholesterol (HDLC) and total cholesterol levels. No significant differences were noted between the groups in alcohol use, lymphocyte count, albumin, hemoglobin, low-density lipoprotein cholesterol (LDLC), or red blood cell (RBC) count (Table 2).

Nutritional scores and BMI in association with CAS

Median GNRI/NRI (94.4 vs. 92.3; p = 0.006) and BMI (23.5 vs. 23.1; p = 0.01) values were modestly but significantly higher in the significant CAS group. However, CONUT and PNI scores, analyzed as either continuous or categorical variables, showed no significant differences between CAS groups. Categorical distributions of nutritional indices and BMI were largely similar between groups, with the exception of GNRI/NRI, which showed a significant difference (Tables 2, 3).

**Table 3:** Baseline characteristics of categories of nutritional scores and BMI Data are presented as n (%). P-values < 0.05 were considered statistically significant. BMI: Body mass index; CAS: Coronary artery stenosis; CONUT: Controlling nutritional status; GNRI: Geriatric nutritional risk index; NRI: Nutritional risk index; PNI: Prognostic nutritional index.

	Significant CAS (n = 503)	Insignificant CAS (n = 604)	χ² value (df)	p-value
BMI			4.43 (2)	0.1
Normal BMI (18.5-24.9)	297 (45.1)	361 (54.9)		
Underweight BMI (<18.49)	35 (36.5)	61 (63.5)		
High BMI (>25)	171 (48.4)	182 (51.6)		
CONUT standard cutoff points			2.58 (3)	0.5
No risk	171 (42.3)	233 (57.67)		
Mild risk	246 (47)	278 (53)		
Moderate risk	78 (47.8)	85 (52.2)		
Severe risk	8 (50)	8 (50)		
NRI/GNRI standard cutoff points			9.96 (3)	0.02
No risk	202 (48)	219 (52)		
Mild risk	93 (51.4)	88 (48.6)		
Moderate risk	76 (46.3)	88 (53.7)		
Severe risk	132 (38.7)	209 (61.3)		
PNI standard cutoff points			0.49 (2)	0.8
No risk	427 (45.3)	516 (54.7)		
Moderate risk	35 (43.7)	45 (56.3)		
Severe risk	41 (48.8)	43 (51.2)		
CONUT (75^th^ percentile) categorization			0.08 (1)	0.8
Low CONUT	366 (45.2)	444 (54.8)		
High CONUT	137 (46.1)	160 (53.9)		
NRI/GNRI (75^th^ percentile) categorization			0.90 (1)	0.3
Low NRI/GNRI	153 (47.7)	168 (52.3)		
High NRI/GNRI	350 (44.5)	436 (55.5)		
PNI (75^th^ percentile) categorization			0.07 (1)	0.8
Low PNI	124 (44.8)	153 (55.2)		
High PNI	379 (45.7)	451 (54.3)		
CONUT (75^th^ percentile) + BMI categorization			6.42 (5)	0.3
Low CONUT + Normal BMI	204 (43.8)	262 (56.2)		
Low CONUT + Underweight BMI	17 (34)	33 (66)		
Low CONUT + High BMI	145 (49.3)	149 (50.7)		
High CONUT + Normal BMI	93 (48.4)	99 (51.6)		
High CONUT + Underweight BMI	18 (39.1)	28 (60.9)		
High CONUT + High BMI	26 (44.1)	33 (55.9)		
NRI/GNRI (75^th^ percentile) + BMI categorization			5.20 (5)	0.4
High NRI/GNRI + Normal BMI	84 (45.2)	102 (54.8)		
High NRI/GNRI + Underweight BMI	1 (50)	1(50)		
High NRI/GNRI + High BMI	68 (51.1)	65 (48.9)		
Low NRI/GNRI + Normal BMI	213 (45.1)	259 (54.9)		
Low NRI/GNRI + Underweight BMI	34 (36.2)	60 (63.8)		
Low NRI/GNRI + High BMI	103 (46.8)	117 (53.2)		
PNI (75^th^ percentile) + BMI categorization			10.39 (5)	0.06
High PNI + Normal BMI	59 (39.1)	92 (60.9)		
High PNI + Underweight BMI	7 (58.3)	5 (41.7)		
High PNI + High BMI	58 (50.9)	56 (49.1)		
Low PNI + Normal BMI	238 (46.9)	269 (53.1)		
Low PNI + Underweight BMI	28 (33.3)	56 (66.7)		
Low PNI + High BMI	113 (47.3)	126 (52.7)		

Malnutrition classification discrepancies across nutritional indices

**Figure 2:**
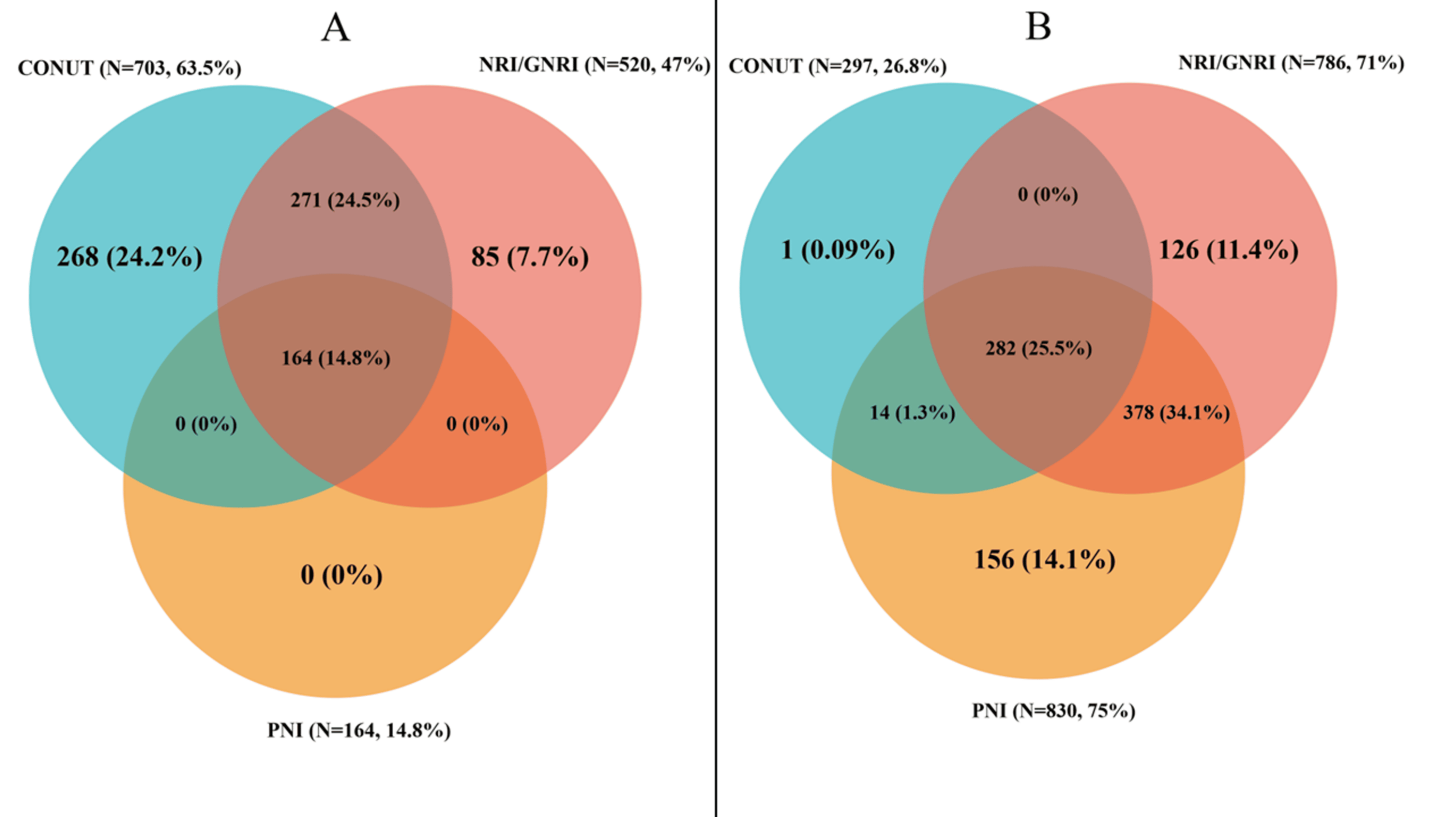
Venn diagrams illustrating the overlap in malnutrition classification among different nutritional assessment tools using (A) standard clinical cutoffs and (B) 75th percentile dichotomization Numbers represent counts of patients classified as malnourished uniquely or commonly by each index (CONUT, GNRI/NRI, and PNI). CONUT: Controlling nutritional status; GNRI: Geriatric nutritional risk index; NRI: Nutritional risk index; PNI: Prognostic nutritional index.

Malnutrition prevalence varied depending on the assessment method. Using standard clinical cutoffs, malnutrition was identified in 63.5% (CONUT), 47% (GNRI/NRI), and 14.8% (PNI) of patients. Only around 15% (n = 164) were classified as malnourished by all three tools, while 28.8% (n = 319) were not identified as malnourished by any index (Figure 2, Panel A). The greatest pairwise overlap was between CONUT and GNRI/NRI (271 patients).

Using the 75th percentile cutoffs, malnutrition prevalence was 26.8% (CONUT), 71% (GNRI/NRI), and 75% (PNI). Concordance improved slightly, with 25.5% (n = 282) classified as malnourished by all tools and 13.6% (n = 150) missed by all (Figure 2, Panel B). The highest pairwise overlap was between GNRI/NRI and PNI (378 patients).

These results demonstrate variability in malnutrition classification depending on the nutritional tool and cutoff applied, highlighting the need for careful tool selection.

Agreement between nutritional indices

Pairwise agreement among the malnutrition classification tools was evaluated using Cohen’s kappa coefficient. Based on standard clinical cutoffs, the agreement was fair between CONUT and GNRI/NRI (κ = 0.371) and between GNRI/NRI and PNI (κ = 0.328), indicating modest concordance. The lowest pairwise agreement was observed between CONUT and PNI (κ = 0.182), reflecting poor concordance. Overall agreement across all three tools, assessed by Fleiss’ kappa, was poor (κ = 0.227), indicating considerable variability in malnutrition classification depending on the tool used (Table 4).

**Table 4:** Agreement among nutritional assessment tools for malnutrition classification using Cohen’s and Fleiss’ kappa coefficients All Cohen’s and Fleiss’ kappa values are statistically significant (p < 0.001). Interpretation of Cohen’s kappa: <0.20 = poor, 0.21-0.40 = fair, 0.41-0.60 = moderate, 0.61-0.80 = good (substantial), and 0.81-1.00 = very good (almost perfect) agreement. Interpretation of Fleiss’ kappa: <0.40 = poor, 0.40-0.75 = intermediate to good, and >0.75 = excellent agreement. CONUT: Controlling nutritional status; GNRI: Geriatric nutritional risk index; NRI: Nutritional risk index; PNI: Prognostic nutritional index.

	Cohen’s Kappa (κ) (95% CI)	% Agree	Interpretation
*Standard clinical cutoffs (any degree of malnutrition)*			
CONUT vs NRI/GNRI	0.372 (0.321-0.424)	68.1	Fair agreement
CONUT vs PNI	0.182 (0.153-0.210)	51.3	Poor agreement
NRI/GNRI vs PNI	0.328 (0.286-0.371)	67.8	Fair agreement
Overall (Fleiss’)	0.227 (0.193-0.261)	-	Poor agreement
*75^th^ percentile cutoffs*			
CONUT vs NRI/GNRI	0.215 (0.181-0.249)	53.1	Fair agreement
CONUT vs PNI	0.215 (0.185-0.245)	51.7	Fair agreement
NRI/GNRI vs PNI	0.323 (0.262-0.384	73.3	Fair agreement
Overall (Fleiss’)	0.168 (0.138-0.202)	-	Poor agreement

When applying the 75th percentile cutoffs, pairwise agreements remained fair, with GNRI/NRI and PNI demonstrating the highest concordance (κ = 0.323). The overall Fleiss’ kappa for all three tools decreased to 0.168, indicating poor agreement and highlighting increased variability in classification at this threshold (Table 4).

Both categorization methods showed poor overall agreement based on Fleiss’ kappa. These findings emphasize the significant influence of the chosen nutritional index and cutoff criteria on malnutrition diagnosis, underscoring inherent inconsistencies among the assessment tools.

Malnutrition prevalence across BMI subgroups by sex

**Figure 3:**
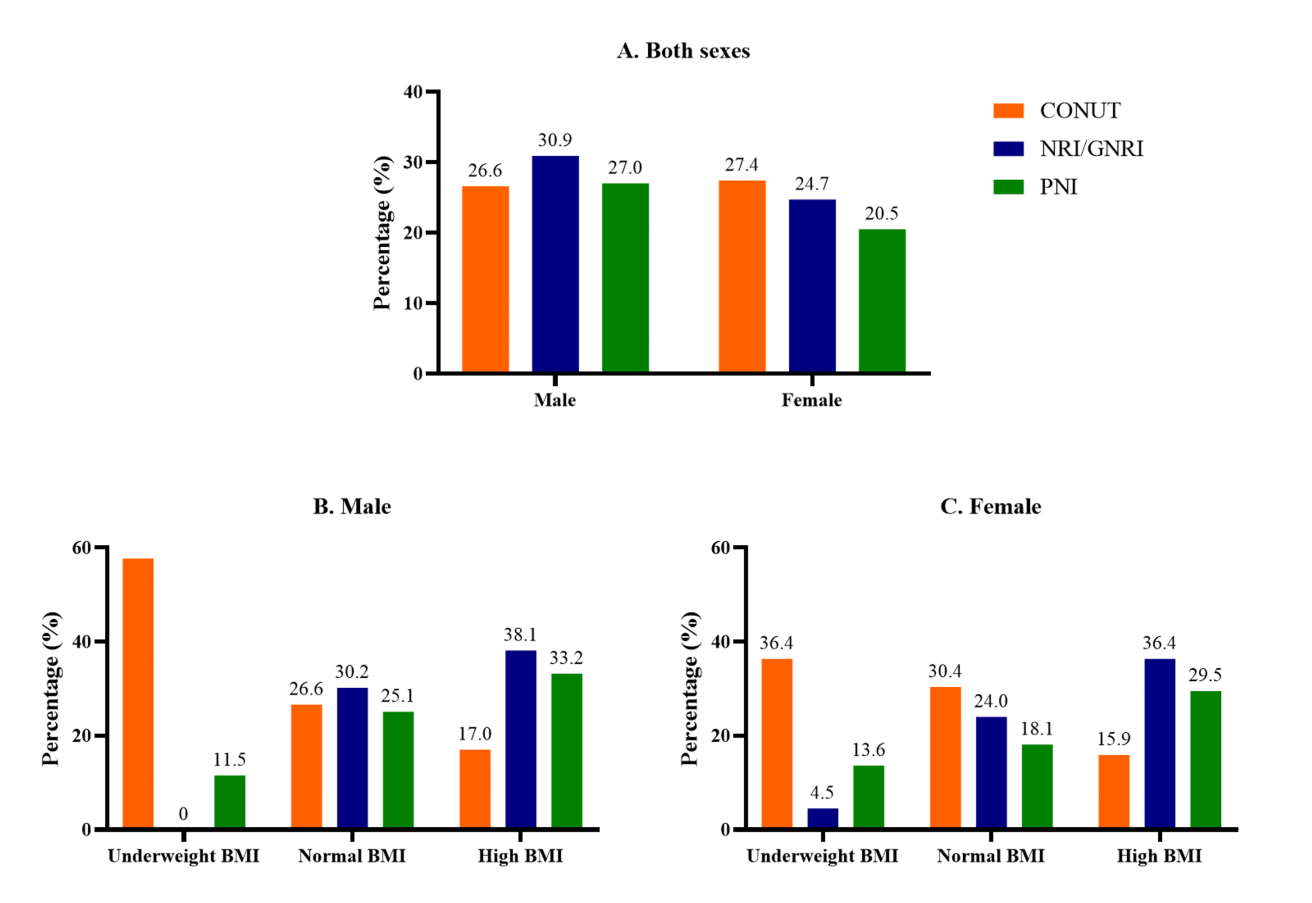
Prevalence of malnutrition by nutritional scores across BMI subgroups by sex (A) Both sexes, (B) male, and (C) female.

Stratification by BMI and sex revealed differing sensitivities of nutritional indices. CONUT detected the highest malnutrition prevalence among underweight individuals (57.7% of males and 36.4% of females), with a decline across normal and high BMI categories. In contrast, GNRI/NRI and PNI identified higher malnutrition prevalence among high BMI individuals, particularly in males (38.1% and 33.2%, respectively) and females (36.4% and 29.5%). These findings highlight index-specific differences in detecting malnutrition depending on body composition and sex (Figure 3).

Univariate predictors of significant CAS

In univariate logistic regression, significant CAS was positively associated with male sex (OR: 1.42; 95% CI: 1.24-1.62), older age (OR: 1.02 per year; 95% CI: 1.01-1.03), and smoking (OR: 1.19; 95% CI: 1.01-1.39). Inflammatory and metabolic markers such as CRP, blood glucose, neutrophil count, WBC count, platelet count, and triglycerides were also significantly associated with CAS (P < 0.05 for all). In contrast, HDLC and total cholesterol showed inverse (protective) associations with CAS.

Among nutritional and anthropometric measures, GNRI/NRI (continuous) was positively associated with CAS (OR: 1.008; 95% CI: 1.003-1.01; P = 0.002), as was BMI (OR: 1.04; 95% CI: 1.005-1.07; P = 0.02). Interestingly, the severe-risk GNRI/NRI category was negatively associated with CAS, while neither CONUT nor PNI categories showed statistically significant associations (Table 5).

**Table 5:** Predictive values for significant CAS by binary logistic regression analysis No significant association was found between any nutritional assessment score and significant coronary stenosis when using standard clinical cutoff categories. However, when interaction with BMI was included in the analysis, notable associations emerged. p < 0.05 was considered statistically significant. ○ indicates variable(s) included in the logistic regression model; × indicates the variable(s) not included in the logistic regression model. BMI: Body mass index; CAS: Coronary artery stenosis; CRP: C-reactive protein; CONUT: Controlling nutritional status; GNRI: Geriatric nutritional risk index; HDLC: High-density lipoprotein cholesterol; LDLC: Low-density lipoprotein cholesterol; NRI: Nutritional risk index; PNI: Prognostic nutritional index; RBC: Red blood cells; WBC: White blood cells.

	Univariate		Multivariate	
	Adjusted OR (95% CI)	p-value	Adjusted OR (95% CI)	p-value
Sex, male	1.42 (1.24-1.62)	<0.0001	1.52 (1.23-1.89)	0.0001
Age	1.02 (1.01-1.03)	0.0005	1.04 (1.02-1.07)	0.0002
Smokers	1.19 (1.01-1.39)	0.03	○	
Drinkers	1.004 (0.88-1.15)	0.9	○	
CRP	1.14 (1.02-1.28)	0.02	○	
Hemoglobin	1.003 (0.95-1.06)	0.9	○	
LDLC	0.99 (0.99-1.001)	0.2	○	
Triglycerides	1.002 (1.0009-1.005)	0.005	1.004 (1.0009-1.007)	0.01
Neutrophil count	1.0001 (1.00008-1.0002)	<0.0001	○	
Platelet count	1.04 (1.02-1.05)	0.0001	1.05(1.01-1.08)	0.004
RBC	1.0003 (0.99-1.002)	0.7	○	
WBC	1.01 (1.005-1.01)	0.0001	○	
Lymphocyte count	0.99 (0.99-1.0001)	0.7	○	
Total cholesterol	0.99 (0.992-0.998)	0.003	○	
HDLC	0.98 (0.96-0.98)	<0.0001	○	
Blood glucose	1.009 (1.005-1.01)	<0.0001	×	
BMI	1.039 (0.006-0.07)	0.02	×	
CONUT score	1.032 (-0.02-0.09)	0.3	×	
NRI/GNRI score	1.008 (0.003-0.013)	0.002	×	
PNI score	0.993 (-0.02-0.01)	0.4	×	
CONUT (vs no risk)			CONUT*BMI interaction	
Mild risk	1.007 (-0.28-0.3)	0.9	-	
Moderate risk	1.044 (-0.3-0.4)	0.8	-	
Severe risk	1.138 (-0.61-0.87)	0.7	-	
NRI/GNRI (vs no risk)			NRI/GNRI*BMI interaction	
Mild risk	1.238 (-0.03-0.46)	0.08	0.87 (0.77-0.97)	0.01
Moderate risk	1.011 (-0.24-0.26)	0.9	-	
Severe risk	0.740 (-0.50 to -0.1)	0.003	1.10 (1.008-1.19)	0.03
PNI (vs no risk)			PNI*BMI interaction	
Moderate risk	0.915 (-0.42-0.24)	0.6	-	
Severe risk	1.122 (-0.21-0.44)	0.5	-	

The combination of low PNI + underweight BMI was inversely associated with CAS (OR: 0.59; 95% CI: 0.38-0.91; P = 0.02), although the subgroup was small (n = 84 total), and findings should be interpreted with caution (Table 6).

**Table 6:** Predictive values of nutritional assessment and BMI categories for significant CAS by univariate regression analysis p < 0.05 was considered statistically significant. BMI: Body mass index; CAS: Coronary artery stenosis; CONUT: Controlling nutritional status; GNRI: Geriatric nutritional risk index; NRI: Nutritional risk index; PNI: Prognostic nutritional index.

	Adjusted OR (95% CI)	p-value
CONUT categories (vs Low CONUT + Normal BMI)		
Low CONUT + Underweight BMI	0.682 (0.41-1.13)	0.1
Low CONUT + High BMI	1.288 (0.99-1.67)	0.053
High CONUT + Normal BMI	1.244 (0.93-1.66)	0.1
High CONUT + Underweight BMI	0.851 (0.51-1.42)	0.5
High CONUT + High BMI	1.043 (0.66-1.64)	0.8
NRI/GNRI categories (vs High NRI/GNRI + Normal BMI)		
High NRI/GNRI + Underweight BMI	1.189 (0.12-12.04)	0.8
High NRI/GNRI + High BMI	1.24 (0.72-2.16)	0.4
Low NRI/GNRI + Normal BMI	0.978 (0.59-1.61)	0.9
Low NRI/GNRI + Underweight BMI	0.674 (0.37-1.21)	0.2
Low NRI/GNRI + High BMI	1.047 (0.62-1.77)	0.9
PNI categories (vs High PNI + Normal BMI)		
High PNI + Underweight BMI	1.653 (0.63-4.34)	0.3
High PNI + High BMI	1.223 (0.84-1.78)	0.3
Low PNI + Normal BMI	1.045 (0.80-1.37)	0.7
Low PNI + Underweight BMI	0.590 (0.38-0.91)	0.02
Low PNI + High BMI	1.059 (0.78-1.44)	0.7
BMI categories (vs Normal BMI)		
Underweight BMI	0.75 (0.56-1.006)	0.054
High BMI	1.23 (1.005-1.5)	0.04

Multivariate models: nutritional indices and BMI interactions

In adjusted multivariate models, no individual nutritional index (CONUT, GNRI/NRI, and PNI) was independently associated with CAS when analyzed using standard categorical cutoffs. However, when interaction terms between BMI (continuous) and nutritional indices were introduced, the severe-risk GNRI/NRI category became significantly associated with higher CAS risk (OR: 1.10; 95% CI: 1.008-1.19). Conversely, the mild-risk GNRI/NRI category was protective (OR: 0.87; 95% CI: 0.77-0.97) (Table 5).

Combined phenotype models further revealed that high CONUT + normal BMI (OR: 1.88; 95% CI: 1.05-3.42) and low CONUT + high BMI (OR: 1.75; 95% CI: 1.05-2.97) were significantly associated with higher CAS odds. Paradoxically, high CONUT + high BMI was inversely associated with CAS (OR: 0.33; 95% CI: 0.10 - 1.01; P = 0.03), though the subgroup was small (n = 59 total), limiting interpretability. In the PNI + BMI model, the high PNI + high BMI group had increased odds of CAS (OR: 1.92; 95% CI: 1.003-3.93; P = 0.04). No significant associations were identified in the GNRI/NRI + BMI model (Table 7).

**Table 7:** Predictive values for significant CAS by multivariate logistic regression analysis p < 0.05 was considered statistically significant. ○ indicates variable(s) included in the logistic regression model; × indicates variable(s) not included in the logistic regression model. BMI: Body mass index; CAS: Coronary artery stenosis; CONUT: Controlling nutritional status; GNRI: Geriatric nutritional risk index; HDLC: High-density lipoprotein cholesterol; LDLC: Low-density lipoprotein cholesterol; NRI: Nutritional risk index; PNI: Prognostic nutritional index.

Variables	CONUT + BMI model		NRI/GNRI + BMI model		PNI + BMI model	
	Adjusted OR (95% CI)	p-value	Adjusted OR (95% CI)	p-value	Adjusted OR (95% CI)	p-value
Sex, male	1.47 (1.16-1.89)	0.001	1.48 (1.20-1.84)	0.0003	1.47 (1.15-1.87)	0.001
Age	1.04 (1.02-1.06)	0.0005	1.04 (1.02-1.06)	<0.0001	1.04 (1.02-1.06)	<0.0001
Smokers	○		○		○	
Drinkers	○		○		○	
CRP	○		○		○	
Hemoglobin	0.70 (0.54-0.90)	0.006	○		0.71 (0.55-0.91)	0.007
LDLC	○		○		○	
Triglycerides	○		1.004 (1.001-1.007)		○	
Neutrophil count	○		○		○	
Platelet count	○		1.04 (1.01-1.08)	0.005	○	
RBC	1.008 (1.0008-1.016)	0.03	○		1.008 (1.0006-1.02)	
WBC	○		○		○	
Blood glucose	1.008(1.003-1.01)	0.002	×		1.008 (1.003-1.01)	0.002
Lymphocyte count	○		○		×	
Total cholesterol	○		○		×	
HDLC	○		○		×	
	CONUT + BMI categories (vs Low CONUT + Normal BMI)		NRI/GNRI + BMI categories (vs High NRI/GNRI + Normal BMI)		PNI + BMI categories (vs High PNI + Normal BMI)	
Low score + Underweight BMI	-		-		-	
Low score + High BMI	1.75 (1.05-2.97)	0.03	-		-	
High score + Normal BMI	1.88 (1.05-3.42)	0.03	-		-	
High score + Underweight	-		-		-	
High score + High BMI	0.33 (0.1003-1.01)	0.03	-		1.92 (1.003-3.93)	0.04

Predictive performance of nutritional indices combined with BMI

Among all combinations, the CONUT + BMI model showed the highest discriminatory ability for CAS, with a C-statistic of 0.71 (95% CI: 0.67-0.75) and the highest sensitivity (78.6%). The PNI + BMI model had the highest PPV at 66.7%, but lower sensitivity (61.3%). The GNRI/NRI + BMI model showed intermediate predictive performance (Table 8). While the CONUT + BMI model demonstrated slightly better performance metrics, none of the models exhibited strong discriminatory power (AUC ≥ 0.80), highlighting the complexity of nutritional risk profiling in the context of CAS.

**Table 8:** Discrimination ability of each malnutrition score for predicting significant CAS p < 0.05 was considered statistically significant. BMI: Body mass index; CAS: Coronary artery stenosis; CONUT: Controlling nutritional status; GNRI: Geriatric nutritional risk index; NRI: Nutritional risk index; PNI: Prognostic nutritional index.

Discrimination ability	CONUT + BMI	NRI/GNRI + BMI	PNI + BMI
Sensitivity, %	78.6	69.6	61.3
Positive predictive value, %	60.9	60.8	66.7
C-statistics (95% CI)	0.71 (0.67-0.75)	0.697 (0.66-0.73)	0.707 (0.67-0.75)
p-value	<0.0001	<0.0001	<0.0001

## Discussion

Prevalence, concordance, and clinical utility of malnutrition indices in CAS

In this retrospective cohort of 1,107 Japanese patients with angiographically confirmed CAS, ranging from insignificant to significant CAS, a key anatomical manifestation of atherosclerosis and a precursor to major adverse cardiovascular events (MACE), CAS served as a clinical window into underlying cardiovascular risk [23,24]. Prevalence ranged from 26.8% using CONUT to over 70% when assessed using PNI and GNRI/NRI when using standard clinical cutoffs. These rates align with international studies: Sze et al. [6] reported malnutrition rates ranging from 8% (PNI) to 54% (CONUT) in heart failure patients. Kalyoncuoğlu et al. [5] found prevalence rates from 6.3% (PNI) to over 60% (GNRI and CONUT) in elderly patients with coronary artery disease (CAD). Similarly, Roubín et al. [7] reported malnutrition prevalence between 50% and 60% among patients with acute coronary syndrome, based on CONUT, PNI, and NRI scores. Importantly, a Japanese study echoes these high burdens: Ishizu et al. reported malnutrition prevalence between 13.8% (PNI) and 60.5% (GNRI) in older patients with severe aortic stenosis undergoing transcatheter aortic valve implantation (TAVI) [14]. Thus, our findings are consistent with both international and Japanese data, reinforcing that malnutrition is prevalent across diverse cardiovascular settings and underscoring the utility of multiple indices in risk evaluation.

Consistent with prior studies, our findings demonstrate considerable discordance in malnutrition classification across different nutritional indices. Roubín et al. [7] reported that only 8.9% of patients were classified as malnourished by all three scores (CONUT, NRI, and PNI), with 28.2% not identified as malnourished by any tool. Similarly, Sze et al. [6] found a 5% concordance rate for malnutrition across CONUT, GNRI, and PNI, with 42% classified as non-malnourished by all. In comparison, our study showed a slightly higher overlap, with 14.8% of patients identified as malnourished by all three tools and 28.8% not classified as malnourished by any index, using standard clinical cutoffs. These findings collectively highlight the limited agreement between nutritional assessment tools, emphasizing their noninterchangeability and the importance of selecting appropriate methods for malnutrition diagnosis, likely reflecting their shared sensitivity to pronounced nutritional deficits. These discrepancies stem from the different physiological domains each tool measures. While CONUT incorporates immune (lymphocyte count) and lipid (total cholesterol) parameters, both potentially influenced by statins [13], GNRI, NRI, and PNI primarily rely on serum albumin and anthropometric measures, thereby better reflecting protein-energy malnutrition and frailty [5,6].

Albumin-based indices (GNRI and NRI) classified a larger proportion of patients as malnourished, particularly among older or metabolically vulnerable individuals, supporting Sze et al.’s recommendation of GNRI as a more comprehensive index in cardiovascular populations. However, GNRI may underestimate malnutrition in overweight individuals, where excess adiposity can obscure sarcopenia and systemic inflammation [6,19].

Serum albumin, common across all indices, is a validated nutritional marker affected by protein intake, insulin activity, and oncotic pressure. Hypoalbuminemia may thus reflect malnutrition, inflammation, or cachexia [13,17]. Chronic inflammation, a key player in atherogenesis, also reduces albumin, which itself exerts anti-inflammatory and antioxidant effects relevant to atherothrombosis. Low lymphocyte counts, integral to CONUT and PNI and predictive of poor CAD outcomes, may signal immune dysfunction from chronic stress. Meanwhile, low cholesterol, traditionally protective, is paradoxically associated with poor outcomes in heart failure, renal dysfunction, and the elderly, supporting the concept of reverse epidemiology [13]. Thus, low albumin, cholesterol, and lymphocyte levels may signal increased risk in heart disease patients.

Crucially, malnutrition was common even among individuals with normal or high BMI, highlighting BMI’s limitations as a standalone nutritional proxy and reinforcing the need for multidimensional tools to detect sarcopenia, inflammation, and cachexia in cardiovascular populations [6,19].

Nutritional status, BMI, and CAS: interactions and insights

Although our study cannot establish a causal relationship between malnutrition and significant CAS, it highlights important associations between malnutrition, BMI as a continuous variable, and CAS risk. Notably, GNRI/NRI remained significantly associated with CAS even after adjusting for BMI interaction, whereas CONUT and PNI did not. A U-shaped association was observed: severe malnutrition was linked to increased CAS risk, while mild malnutrition appeared paradoxically protective. This pattern may reflect the complex interplay of undernutrition and overnutrition in atherogenesis, potentially mediated by inflammation, sarcopenia, and metabolic dysfunction. Our findings are consistent with previous studies. For example, Yildirim et al. [25] reported significant associations between all three indices, CONUT, GNRI, and PNI and long-term mortality in non-ST-elevation myocardial infarction (NSTEMI) patients after PCI, with GNRI showing similar strength of association as CONUT and stronger than PNI. Likewise, Basta et al. [26] found that CONUT, but not PNI, was significantly associated with two-year mortality in elderly ST-elevation myocardial infarction (STEMI) patients. These findings support our results, where only GNRI/NRI showed an independent association with CAS in multivariable models.

Stratifying indices at the 75th percentile and integrating them with BMI yielded further insights. Some phenotypes, e.g., high CONUT + normal BMI, low CONUT + high BMI, or high PNI + high BMI, were associated with elevated CAS risk. Interestingly, high CONUT + high BMI was inversely associated with CAS, though the small sample size tempers interpretation. This finding may reflect survival bias, compensatory metabolic adaptations, or BMI’s inability to distinguish fat from lean mass. Additionally, individuals with obesity and poor nutritional status may receive more frequent medical surveillance or dietary counseling, leading to earlier intervention and cardiovascular risk factor management [27]. Chronic inflammation and altered lipid metabolism in malnourished-obese individuals may also predispose to microvascular or nonobstructive disease rather than significant epicardial stenosis [28]. The “obesity paradox” described in cardiovascular literature may further contribute to this observation [29]. These nuances underscore the emerging risk phenotype of sarcopenic obesity, patients with high BMI and underlying muscle wasting or inflammation [15,16].

Unlike earlier work, such as Kunimura et al. [12], which excluded underweight individuals to avoid reverse causality, our study included them, offering a broader view. We also found a significant association between CRP and CAS, contrasting with Kunimura et al., who only observed a nonsignificant trend between elevated CRP and high CONUT + normal BMI. While we lacked data on advanced inflammatory markers like TNF-α or IL-6, which may mediate the malnutrition-inflammation-atherosclerosis (MIA) pathway, these warrant future study [13].

Sarcopenic obesity, recognized as a high-risk cardiovascular phenotype [18,19], overlaps with the obesity paradox, where overweight individuals sometimes experience better outcomes, possibly due to greater muscle reserves or survivor bias. Similarly, the cholesterol paradox (low cholesterol linked to poor prognosis) may reflect underlying cachexia or advanced disease [13,30].

While GNRI and NRI showed strong associations with CAS in univariate analyses, these weakened when BMI was included, likely due to shared variables (e.g., weight), creating multicollinearity. This divergence from prior studies underscores the challenge of modeling nutritional and anthropometric data jointly.

Clinical and public health implications

To our knowledge, this is the first Japanese coronary angiography study to simultaneously evaluate four nutritional indices: CONUT, PNI, GNRI, and NRI, in combination with BMI, including underweight categories, for their association with angiographically confirmed CAS. While prior Japanese studies have assessed individual or paired indices, such as CONUT in PCI cohorts [13]; CONUT with BMI in stable CAD patients [12]; or CONUT, PNI, and GNRI in TAVI older patients [14], none have integrated all four indices together with BMI within a coronary angiography context. Our finding that the CONUT + BMI profile yielded the highest sensitivity for detecting CAS suggests that combining a biochemical/immune-nutrition score with anthropometric measures enhances cardiovascular risk detection, especially among individuals with normal BMI but poor nutritional status [12].

Clinically, these results support incorporating nutritional indices alongside BMI into routine cardiovascular risk assessments. Different indices may capture distinct physiological dimensions and thus be more applicable in varied clinical settings, for example, CONUT and PNI in acute or immunocompromised patients, and GNRI and NRI in chronic or geriatric populations [5-8,12-14]. Embedding such combined tools into EMR could enable real-time, phenotype-driven cardiovascular alerts, offering a scalable, cost-effective strategy to identify high-risk individuals and facilitate earlier interventions, particularly important in aging populations like Japan’s [12,13,16].

Furthermore, the reliance on widely available clinical parameters such as serum albumin, total cholesterol, lymphocyte count, height, and weight enhances the applicability of this approach in LMICs where advanced diagnostic resources may be scarce [3,4]. Nations facing a dual burden of malnutrition and cardiovascular disease, such as India, Nigeria, and Indonesia, could particularly benefit from integrating these nutritional indices with BMI into primary care workflows. This combined strategy offers a practical, low-cost, and scalable framework for cardiovascular risk stratification in resource-limited settings, helping to address global health disparities [2].

Strengths, limitations, and future directions

A notable strength of this study is the use of two complementary analytic strategies: standard clinical cutoffs with BMI modeled as a continuous interaction term in logistic regression and phenotype-based combinations using dichotomized nutritional indices (75th percentile) with categorical BMI groups (underweight, normal, and high). This dual approach allowed for both nuanced and practical risk profiling.

However, several limitations warrant consideration. First, the retrospective, cross-sectional, single-center design limits causal inference, generalizability, and the ability to capture temporal changes in nutritional status or CAD progression. Second, the absence of longitudinal follow-up prevents evaluation of long-term clinical outcomes such as MACE or mortality. Third, potential selection bias cannot be excluded, as patients undergoing coronary angiography may not represent the broader population with or at risk for CAD.

Fourth, although we focused on simple and accessible biomarkers, we acknowledge that important cardiometabolic confounders, such as renal function, oxidative stress markers, vascular function, cardiac injury/stress markers, dietary intake, inflammatory status, statin therapy, diabetes, and hepatic dysfunction, were not available and may influence both nutritional status and CAS risk. Future studies incorporating these parameters could provide more comprehensive risk stratification.

Fifth, BMI was used as a measure of body composition, but it cannot differentiate fat from lean mass or reflect central adiposity. Incorporation of waist circumference or other measures of body fat distribution would enhance assessment, especially considering the potential impact of sarcopenic obesity and exercise on cardiovascular risk.

Finally, our endpoint was angiographically detected CAS rather than clinical outcomes. While CAS is a valid surrogate for cardiovascular risk, prospective studies are needed to confirm prognostic implications using definitive endpoints. Additionally, while 75th percentile cutoffs used for some analyses align with prior research, such thresholds may limit comparability across studies and require validation in independent cohorts.

## Conclusions

Our findings suggest that GNRI and NRI, when interacted alongside BMI (continuous), provide a sensitive gradient of nutritional risk related to CAS. Meanwhile, percentile-based combinations of CONUT or PNI with categorical BMI values offer useful categorical risk phenotypes. Together, these indices, especially when paired with BMI, provide a nuanced approach to cardiovascular risk stratification, capable of detecting vulnerability even in patients without overt obesity or cachexia.

In clinical and public health contexts, especially in LMICs, the use of such accessible and objective indices holds strong potential for early identification of at-risk individuals. Future prospective studies should assess whether interventions guided by these indices can improve cardiovascular outcomes. Further integration with inflammatory markers, frailty scores, and body composition assessments may enhance the predictive power of these models and better inform preventive strategies.
